# Supplementary figures and images for: Slope and distance from buildings are easy-to-retrieve proxies for estimating livestock site-use intensity in alpine summer pastures
Source: PLoS One. 2021 Nov 3;16(11):e0259120. doi: 10.1371/journal.pone.0259120 (PMC8565746; doi:10.1371/journal.pone.0259120)

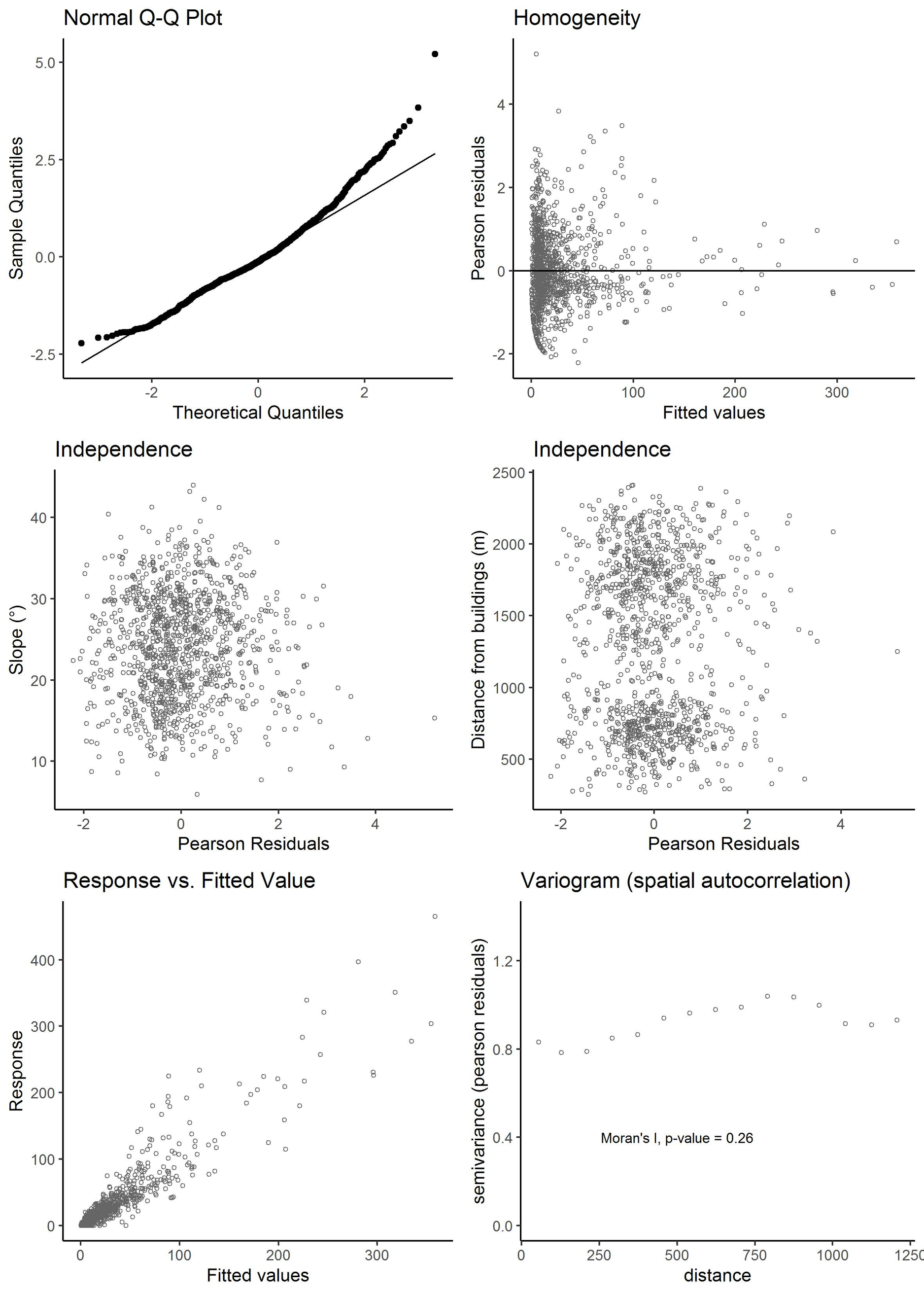

Supplement: S1 Fig — (TIF) [file pone.0259120.s001.tif]
