# Supplementary material for: Slope and distance from buildings are easy-to-retrieve proxies for estimating livestock site-use intensity in alpine summer pastures
Source: PLoS One. 2021 Nov 3;16(11):e0259120. doi: 10.1371/journal.pone.0259120 (PMC8565746; doi:10.1371/journal.pone.0259120)
Supplement: Attachment — Submitted filename: Response to Reviewers.docx [file pone.0259120.s003.docx]

PONE-D-21-19475
Slope and distance from buildings are easy-to-retrieve proxies for estimating livestock site-use intensity in alpine summer pastures
PLOS ONE

Journal requirements:

When submitting your revision, we need you to address these additional requirements.

1. Please ensure that your manuscript meets PLOS ONE's style requirements, including those for file naming. The PLOS ONE style templates can be found at

<https://journals.plos.org/plosone/s/file?id=wjVg/PLOSOne_formatting_sample_main_body.pdf> and

<https://journals.plos.org/plosone/s/file?id=ba62/PLOSOne_formatting_sample_title_authors_affiliations.pdf>

We modified the manuscript style accordingly

2. In your Methods section, please provide additional information regarding the permits you obtained for the work. Please ensure you have included the full name of the authority that approved the field site access and, if no permits were required, a brief statement explaining why.

We specified in Methods section that all the permissions and permits required for fieldwork and utilization of cattle were requested and authorized by the administration of Val Troncea Natural Park.

3. In your Methods section, please provide additional details regarding participant consent from the owners of the animals. In the ethics statement in the Methods and online submission information, please ensure that you have specified (1) whether consent was informed and (2) what type you obtained (for instance, written or verbal). If the need for consent was waived by the ethics committee, please include this information.

We specified in Methods section that in agreement with the farmer 14 cows were randomly selected for being tracked with GPS collars.

4. We note that you have stated that you will provide repository information for your data at acceptance. Should your manuscript be accepted for publication, we will hold it until you provide the relevant accession numbers or DOIs necessary to access your data. If you wish to make changes to your Data Availability statement, please describe these changes in your cover letter and we will update your Data Availability statement to reflect the information you provide

We specified in the submission form, at the “Additional information” section, at the point “Describe where the data may be found in full sentences. If you are copying our sample text, replace any instances of XXX with the appropriate details.” that the data that support the findings of this study are available from the corresponding author upon reasonable request.

5. We note that Figure 1 in your submission contain map images which may be copyrighted. All PLOS content is published under the Creative Commons Attribution License (CC BY 4.0), which means that the manuscript, images, and Supporting Information files will be freely available online, and any third party is permitted to access, download, copy, distribute, and use these materials in any way, even commercially, with proper attribution. For these reasons, we cannot publish previously copyrighted maps or satellite images created using proprietary data, such as Google software (Google Maps, Street View, and Earth). For more information, see our copyright guidelines: <http://journals.plos.org/plosone/s/licenses-and-copyright>.

We require you to either (1) present written permission from the copyright holder to publish these figures specifically under the CC BY 4.0 license, or (2) remove the figures from your submission:

  a. You may seek permission from the original copyright holder of Figure 1 to publish the content specifically under the CC BY 4.0 license.

We recommend that you contact the original copyright holder with the Content Permission Form (<http://journals.plos.org/plosone/s/file?id=7c09/content-permission-form.pdf>) and the following text:

“I request permission for the open-access journal PLOS ONE to publish XXX under the Creative Commons Attribution License (CCAL) CC BY 4.0 (<http://creativecommons.org/licenses/by/4.0/>). Please be aware that this license allows unrestricted use and distribution, even commercially, by third parties. Please reply and provide explicit written permission to publish XXX under a CC BY license and complete the attached form.”

Please upload the completed Content Permission Form or other proof of granted permissions as an "Other" file with your submission.

In the figure caption of the copyrighted figure, please include the following text: “Reprinted from [ref] under a CC BY license, with permission from [name of publisher], original copyright [original copyright year].”

  b. If you are unable to obtain permission from the original copyright holder to publish these figures under the CC BY 4.0 license or if the copyright holder’s requirements are incompatible with the CC BY 4.0 license, please either i) remove the figure or ii) supply a replacement figure that complies with the CC BY 4.0 license. Please check copyright information on all replacement figures and update the figure caption with source information. If applicable, please specify in the figure caption text when a figure is similar but not identical to the original image and is therefore for illustrative purposes only.

The following resources for replacing copyrighted map figures may be helpful:

USGS National Map Viewer (public domain): <http://viewer.nationalmap.gov/viewer/>

The Gateway to Astronaut Photography of Earth (public domain): <http://eol.jsc.nasa.gov/sseop/clickmap/>

Maps at the CIA (public domain): <https://www.cia.gov/library/publications/the-world-factbook/index.html> and <https://www.cia.gov/library/publications/cia-maps-publications/index.html>

NASA Earth Observatory (public domain): <http://earthobservatory.nasa.gov/>

Landsat: <http://landsat.visibleearth.nasa.gov/>

USGS EROS (Earth Resources Observatory and Science (EROS) Center) (public domain): [http://eros.usgs.gov/#](http://eros.usgs.gov/)

Natural Earth (public domain): <http://www.naturalearthdata.com/>

The image on the left has the digital surface model (DSM) of the European Environment Agency (EEA) as background. At the following link https://www.eea.europa.eu/data-and-maps/data/copernicus-land-monitoring-service-eu-dem at the point of Rights is reported: “*Access to data is based on a principle of full, open and free access as established by the Copernicus data and information policy Regulation (EU) No 1159/2013 of 12 July 2013. This regulation establishes registration and licensing conditions for GMES/Copernicus users. Free, full and open access to this data set is made on the conditions that:*

*1.When distributing or communicating Copernicus dedicated data and Copernicus service information to the public, users shall inform the public of the source of that data and information.”*

The image on the right is a map available from the repository of the Piemonte Region (http://www.geoportale.piemonte.it/geocatalogorp/?sezione=catalogo) and it is covered by the Creative Commons license (see http://www.datigeo-piem-download.it/direct/Geoportale/RegionePiemonte/Licenze/New/Licenza_CC40BY.pdf)

According to these indications we have reported in the legend of Figure 1 the sources of the images used as backgrounds.

6. Please review your reference list to ensure that it is complete and correct. If you have cited papers that have been retracted, please include the rationale for doing so in the manuscript text, or remove these references and replace them with relevant current references. Any changes to the reference list should be mentioned in the rebuttal letter that accompanies your revised manuscript. If you need to cite a retracted article, indicate the article’s retracted status in the References list and also include a citation and full reference for the retraction notice.

We do not have any retracted article

Reviewers' comments:

Reviewer's Responses to Questions

**Comments to the Author**

1. Is the manuscript technically sound, and do the data support the conclusions?

The manuscript must describe a technically sound piece of scientific research with data that supports the conclusions. Experiments must have been conducted rigorously, with appropriate controls, replication, and sample sizes. The conclusions must be drawn appropriately based on the data presented.

Reviewer #1: Yes

2. Has the statistical analysis been performed appropriately and rigorously?

Reviewer #1: I Don't Know

3. Have the authors made all data underlying the findings in their manuscript fully available?

The [PLOS Data policy](http://www.plosone.org/static/policies.action#sharing) requires authors to make all data underlying the findings described in their manuscript fully available without restriction, with rare exception (please refer to the Data Availability Statement in the manuscript PDF file). The data should be provided as part of the manuscript or its supporting information, or deposited to a public repository. For example, in addition to summary statistics, the data points behind means, medians and variance measures should be available. If there are restrictions on publicly sharing data—e.g. participant privacy or use of data from a third party—those must be specified.

Reviewer #1: Yes

4. Is the manuscript presented in an intelligible fashion and written in standard English?

PLOS ONE does not copyedit accepted manuscripts, so the language in submitted articles must be clear, correct, and unambiguous. Any typographical or grammatical errors should be corrected at revision, so please note any specific errors here.

Reviewer #1: Yes

5. Review Comments to the Author

Please use the space provided to explain your answers to the questions above. You may also include additional comments for the author, including concerns about dual publication, research ethics, or publication ethics. (Please upload your review as an attachment if it exceeds 20,000 characters)

Reviewer #1: General assessment:

The paper provides interesting information in the field of grazing pressure in alpine pastures. The subject falls within the general scope of the PLOSONE journal. The originality of the work’s contribution lies in demonstrating that slope and distance to buildings along with the types of vegetation and the use of GPS collars contribute to explain the use of grasslands. The methodology is adequate. The interpretations are well justified and consistent with the objectives.
Listed below are several minor aspects that can clarify the article. With attention to these points the paper should be acceptable.

Specific points:

- Remove double parentheses in lines 54, 82, 90, 110, 111 and 146.

We modified the text accordingly

- Line 66. There are some other works that used low-cost GPS collars (e.g. Hanpson et al 2010. Monitoring distances travelled by horses using GPS tracking collars. Australian Veterinary Journal).

Thank you! We added the reference you suggested and also others related to the use of low-cost GPS collars (26 – 29). These research have been carried out in USA and Australian rangelands. Therefore, we modified the text accordingly: “Moreover, up to date, low-cost GPS have been tested only in USA [25-28] and Australian rangelands [29]) and information about their performance is still unavailable in rough environments such as alpine pastures.”

26.	Brennan, J., Johnson, P., & Olson, K. Classifying season long livestock grazing behavior with the use of a low-cost GPS and accelerometer. Computers and Electronics in Agriculture, 2021. 181, 105957.

27.	Karl, J. W., & Sprinkle, J. E. Low-cost livestock global positioning system collar from commercial off-the-shelf parts. Rangeland Ecology & Management, 2019. 72(6), 954-958.

28.	McGranahan, D. A., Geaumont, B., & Spiess, J. W. Assessment of a livestock GPS collar based on an open‐source datalogger informs best practices for logging intensity. Ecology and evolution, 2018. 8(11), 5649-5660.

29.	Hampson, B. A., Morton, J. M., Mills, P. C., Trotter, M. G., Lamb, D. W., & Pollitt, C. C. Monitoring distances travelled by horses using GPS tracking collars. Australian Veterinary Journal, 2010. 88(5), 176-181.

- Lines 74-77. Authors must provide a bibliographic citation that accredits this sentence.

Two citations (29 and 30) are provided in the next sentence, in which we highlight that many authors found a strong relationship between the site use intensity gradient and the transition of specific vegetation communities.

29. Peter M, Edwards PJ, Jeanneret P, Kampmann D, Lüscher A. Changes over three decades in the floristic composition of fertile permanent grasslands in the Swiss Alps. Agric Ecosyst Environ 2008; 125(1–4):204–12.

30. Tasser E, Tappeiner U. Impact of land use changes on mountain vegetation. Appl Veg Sci. 2002;5(2):173–84.

- Lines 103-104. Authors should detail the types of buildings considered.

In the introduction, we specified as examples the farm centers and cowshed, whereas in the Material and Methods (i.e. ‘Computation of site-use intensity proxies’ section) we also indicated ‘other buildings related to pastoral activities’ which are buildings useful as a foothold for pastoral equipment (e.g. mobile electric fences, posts, energizers).

- Lines 120-121. This sentence requires a citation too.

We did not add a citation as in the previous paragraph (from L73 to L86) we discussed, with several references, about the relations between livestock site-use intensity and botanical composition.

- Line 182. Clarify if the distance considered was in a straight line or following the available paths.

We added a sentence to better explain the concept that: “Proximity was based on a straight-line distance between vegetation surveys and buildings.”

- Line 187. Clarify if for the calculation of the stocking rate only the animal that carried the GPS or the entire herd was considered.

Thank you for your comment. Actually, in this analysis we did not aim at calculating the stocking rate, but rather at assessing if slope and distance from buildings were good predictors of site-use intensity, measured by the number of GPS locations. As the term ‘stocking rate’ may be misleading in this context, we removed it from the sentence.
